# Fluid-solid coupling model and biological features of large vestibular aqueduct syndrome

**DOI:** 10.3389/fbioe.2023.1106371

**Published:** 2023-05-11

**Authors:** Zewen Chen, Mengjie Luo, Can Zhou, Xu Bie, Shen Yu, Xiuzhen Sun

**Affiliations:** ^1^ Department of Otolaryngology, Head and Neck Surgery, The Second Affiliated Hospital of Dalian Medical University, Dalian, China; ^2^ State Key Laboratory of Structural Analysis for Industrial Equipment, Dalian University of Technology, Dalian, China

**Keywords:** large vestibular aqueduct syndrome (LVAS), vestibular aqueduct (VA), biomechanics, fluid solid coupling, sensorineural deafness

## Abstract

**Objective:** Computed tomography (CT) images of the temporal bone of large vestibular aqueduct syndrome (LVAS) patients were used to establish 3D numerical models based on the structure of the inner ear, which are, in turn, used to construct inner ear fluid-solid coupling models. The physiological features and pathophysiology of LVAS were analyzed from a biomechanical perspective using finite element analysis.

**Methods:** CT images of the temporal bone were collected from five children attending the Second Hospital of Dalian Medical University in 2022. The CT images were used to build 3D models of the inner ear containing the vestibular aqueduct (VA) by Mimics and Geomagic software, and round window membrane models and fluid-solid coupling models were built by ANSYS software to perform fluid-solid coupling analysis.

**Results:** By applying different pressure loads, the deformation of the round window membranes occurred, and their trend was basically the same as that of the load. The deformation and stress of the round window membranes increased with the increase in load. Under the same load, the deformation and stress of the round window membranes increased with the expansion of the midpoint width of the VA.

**Conclusion:** CT images of the temporal bone used clinically could establish a complete 3D numerical model of the inner ear containing VA. Fluctuations in cerebrospinal fluid pressure could affect inner ear pressure, and VA had a limiting effect on the pressure from cerebrospinal fluid. The larger the VA, the smaller the limiting effect on the pressure.

## 1 Introduction

The vestibular aqueduct (VA) is a bony channel inside the temporal bone, with an average length of 8.7 mm and an overall “J” shape (Wilbrand et al., 1974; Hirai et al., 2006). The VA is lined by the endolymphatic duct, and the inner opening of the VA is located in the vestibule, while the outer opening is located at the intersection between the endolymphatic duct and endolymphatic sac, allowing the endolymphatic duct to connect to the vestibule and endolymphatic sac (Kelemen, 1976; Valvassori and Clemis, 1978). The endolymphatic duct plays an important role in eliminating endolymphatic metabolites and maintaining the stability of endolymphatic metabolism (Ferster et al., 2017; Mendenhall et al., 2018). Therefore, VA is also considered an important structure for maintaining the stability of lymph metabolism in the inner ear.

At a young age, children with VA enlargement frequently develop progressive and fluctuating sensorineural hearing loss with vertigo and tinnitus, especially after head trauma (Lai and Shiao, 2004; Zalewski et al., 2015). The cause of these symptoms is still unclear. Such enlarged VA accompanied by hearing loss is known as large vestibular aqueduct syndrome (LVAS) (Lai and Shiao, 2004; Ruthberg et al., 2019). Valvassori first named this congenital disorder in 1978 and proposed certain diagnostic criterium (width >1.5 mm at the midpoint) (Valvassori and Clemis, 1978). Boston et al. suggested more sensitive criteria to diagnose LVA (width >0.9 mm at the midpoint or >1.9 mm at the operculum) in 2007 (Vijayasekaran et al., 2007).

There are many different theories about the causes of hearing loss due to LVAS. Pressure wave theories suggest that an enlarged VA could allow for transmitting greater intracranial pressure to the inner ear, thereby damaging the inner ear (Okamoto et al., 1998; Naganawa et al., 2000; Gopen et al., 2011). Electrolyte imbalance theory suggests that an enlarged and dysfunctional endolymphatic sac leads to electrolyte derangement and damages the inner ear (Jackler and La Cruz, 1989; Eckhard et al., 2020). Similar to the electrolyte imbalance theory is the hyperosmolar fluid reflux theory, which suggests that hyperosmolar fluid in the endolymphatic sac could enter the inner ear through the enlarged VA, thus, causing damage to the inner ear (Schuknecht and Richter, 1980; Okamoto et al., 1998). Third-window lesion theory suggests that the abnormal opening in the bony labyrinth results in sound energy being shunted out of the cochlea and an enhancement in bone conduction, leading to the characteristic mixed hearing loss seen in many cases (Merchant et al., 2007).

The VA is a precise structure, and the size of the VA is mainly assessed by computed tomography (CT) images, but the observation of its function and overall morphology remains poor. In contrast, most current studies tend to use tissue sections, animal models, and cadaver heads for the observation of VA structures. However, the above-mentioned methods are complicated and costly. Therefore, 3D models of the inner ear of LVAS patients using CT images of the temporal bone become a more suitable option.

Biomechanical finite element analysis has gradually come to the attention of researchers in recent years. The finite element formulation could be applied to various fields, such as heat transfer, torsion of elastic materials, diffusion, and fluid flow, providing researchers with a whole new perspective (Pang et al., 2021). While the inner ear is mainly liquid, its interaction with the surrounding tissue provides the possibility of finite element fluid-solid coupling analysis (Han et al., 2020).

Currently, there are few studies on the finite element analysis of LVAS, and the pathophysiology of LVAS is highly controversial among researchers. In this study, we used CT images of the temporal bone to reconstruct the inner ear model. Through finite element fluid-solid coupling calculation, we analyzed the biomechanical characteristics of the inner ear of LVAS patients and normal people and then explained the pathophysiological mechanism of LVAS from the perspective of biomechanics.

## 2 Materials and methods

### 2.1 Materials

We obtained temporal bone CT images from five patients who attended the Second Hospital of Dalian Medical University in 2022. Four children were confirmed to have LVAS according to the Cincinnati criteria (width >0.9 mm at the midpoint or width >1.9 mm at the operculum), and the other child had normal inner ear development. Five patients ranged in age from 1 to 10 years, with a mean age of 4 years. Of the four children with LVAS, three had the disease in both ears, and one had the disease in one ear. None patients had undergone any ear surgery. Of the 4 children, 2 had prior experience with vertigo, and the other 2 had no obvious vestibular symptoms. This study was approved by the Ethics Committee of the Second Affiliated Hospital of Dalian Medical University.

### 2.2 CT measurement

All patients were scanned with a Philips 128-row CT, and the scan area was from the superior edge of the temporal bone to the inferior edge of the external auditory canal foramen, with a slice thickness of 1 mm. The width of the midpoint and operculum of the VA was measured in the axial view of all patients bilaterally, respectively.

### 2.3 3D modeling

The CT images in DICOM format were imported into Mimics 15.0, and the VA midpoint and operculum width were measured. The inner ear models containing the VA were created from the CT images. Then, the established models were imported into Geomagic 12.0 in STL format, and operations such as smoothing, denoising, meshing, and surface fitting were performed to obtain smooth models of the preliminary mesh. Finally, referring to the normal anatomy of the inner ear, we located the position of the round window membrane at the lower part of the promontory and the lower part of the vestibular window in ANSYS 15.0. Since there was no significant difference between the bilateral inner ears of the normal child, the right inner ear was selected to establish a normal model.

### 2.4 Numerical simulation and parameter setting

We meshed the inner ear and round window membrane models and verified the mesh validity. We assumed that the inner ear model, except for the round window membrane, is full of lymph and set it as a fluid region, while the round window membrane model was set as a solid region.

### 2.5 Fluid area condition setting

We calculated the state by Reynolds number (Re). The calculation method was transient calculation. Dynamic viscosity $\left(\mu \right):1\times 10^{-3}\;Pa\;\cdot \;s$ ;

Density (ρ): 1,000 kg/m^3^. Since Re = ρvd/μ , when d = 1 mm,

$$Re=1000\times 0.00547\times 0.00086/1\times 10^{-3}\approx 5$$

In this case, the effect of viscosity is greater than the inertial force; hence, the fluid should be laminar, and the laminar flow equation is applied to the fluid-solid coupling calculation.

### 2.6 Solid area condition setting

The following settings were used to establish solid area condition: elastic film property of 3 MPa, Poisson’s ratio of 0.3, and thickness of 60 nm.

### 2.7 Pressure setting

Previous studies have found that when the human head is subjected to external forces, the pressure of cerebrospinal fluid rises to 0.5–300 kPa (Cao et al., 2016). Thus, the inlet pressures were set to different sine functions. The maximum pressures were 300 kPa, 250 kPa, 200 kPa, 150 kPa, 100 kPa, and 10 kPa. The load time was set to 0.012 s, where the load reached its maximum value at 0.006 s, and the observation time was set to 0.025 s.

### 2.8 Operational equation.

Ansys 15.0 was used to calculate the interaction between the inner ear lymph and the round window membrane under the action of external forces.

The main control equations of the fluid model were:

$$\rho_{f}\frac{\partial v_{i}}{\partial x_{i}}=0$$

$$\rho_{f}\frac{\partial v_{i}}{\partial t}+\rho_{f}v_{j}\frac{\partial v_{i}}{\partial x_{j}}=-\frac{\partial p}{\partial x_{i}}+\frac{\partial \tau_{ij}}{\partial x_{j}}+\rho_{f}f_{i}$$

$$\tau_{ij}=\mu \left(\frac{\partial v_{i}}{\partial x_{j}}+\frac{\partial v_{j}}{\partial x_{i}}\right)$$

Here, fluid density is represented by $\rho_{f}$ , fluid pressure is represented by p , and velocity vector is represented by $v_{i}$ .

Equations of motion for the solid model were:

$$\rho_{s}\frac{\partial^{2}u_{i}}{\partial t^{2}}=\frac{\partial \sigma_{ij}^{s}}{\partial x_{j}}+g_{i}$$

$$\sigma_{ij}^{s}=\frac{Ev}{\left(1+v\right)\left(1-2v\right)}\varepsilon_{kk}\delta_{ij}+\frac{E}{\left(1+v\right)}\varepsilon_{ij}$$

$$\varepsilon_{ij}=\frac{1}{2}\left(\frac{\partial u_{i}}{\partial x_{j}}+\frac{\partial u_{j}}{\partial x_{i}}\right)$$

Here, the stress tensor of the solid is represented by $\sigma_{ij}^{s}$ , displacement vector of the solid is represented by $u_{i}$ , and strain tensor is represented by $\varepsilon_{ij}$ .

The motion continuity condition (the normal velocity at the liquid-solid interface remained continuous) and the force continuity condition needed to be satisfied at the flow-solid interface.

$$u^{s}=u^{f}$$

$$v^{s}=v^{f}$$

$$\sigma^{s}*n=\sigma^{f}*n$$

Here, displacement of the solid interface is represented by $u^{s}$ , displacement of the fluid interface is represented by $u^{f}$ , the velocity of the fluid interface is represented by $v^{f}$ , velocity of the solid interface is represented by $v^{s}$ , and the normal direction of the fluid-solid interface is represented by n .

### 2.9 Fluid-solid coupling model setup

We set the area in the inner ear model that was in contact with the round window membrane as dynamic mesh. The part of the VA in contact with the endolymphatic sac was set as the pressure inlet, and all other areas were set as the wall. Since the flow of lymph in the inner ear is small, we assumed that the fluid area was closed; therefore, there was no pressure outlet. The part of the round window membrane model in contact with the inner ear model was set as a fluid-solid interface, and the round window membrane was fixed around it to prevent its displacement. All models had the above-mentioned settings.

## 3 Results

### 3.1 Vestibular aqueduct measurement data

Table 1 shows the results of measuring the midpoint and operculum widths of all inner ear VAs. The inner ears of all patients met the Cincinnati criteria. Inner ears with LVAS (A–G) were sorted and named by VA midpoint width.

**TABLE 1:** Width of external mouth and the midpoint of the vestibular aqueduct in different models.

Inner ear	Width of operculum (mm)	Width of midpoint (mm)
Normal	1.2	0.8
A	3.6	2.2
B	3.4	2.2
C	3.2	2.25
D	3.7	2.3
E	3.6	2.4
F	4.6	2.8
G	4.3	3.3

### 3.2 3D modeling and meshing of the inner ear

**FIGURE 1:**
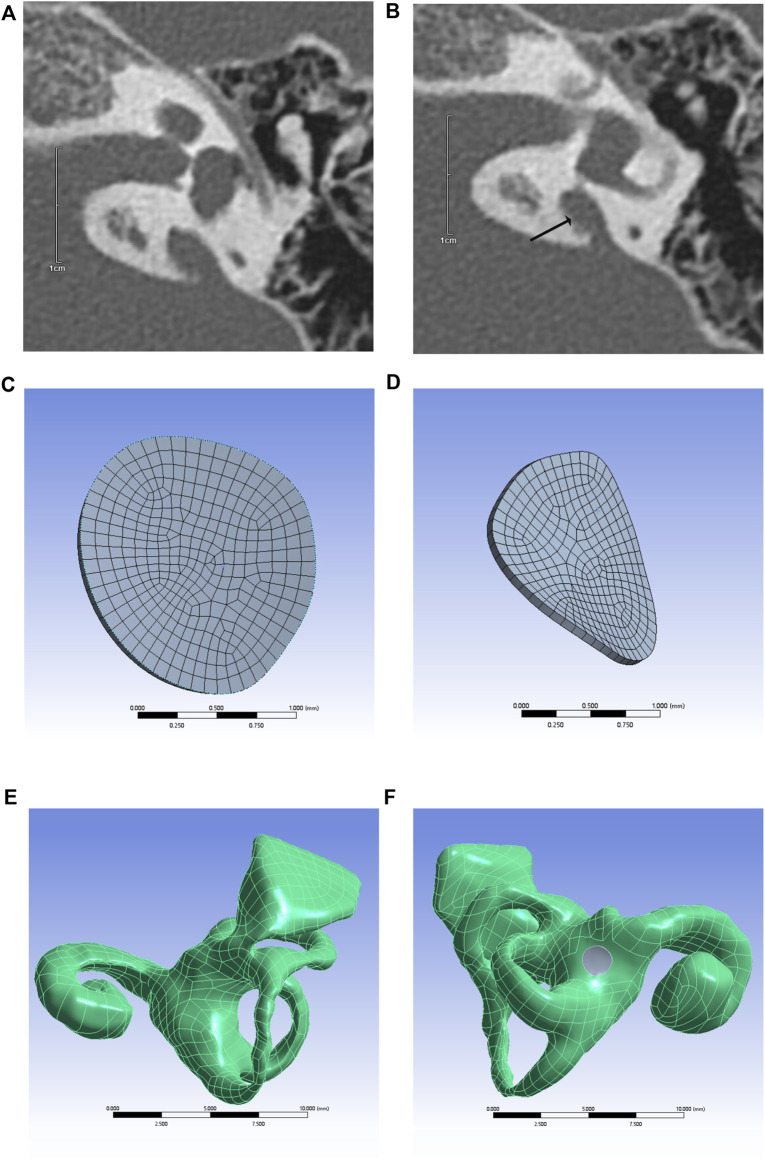
CT image of temporal bone with enlarged VA (arrows) **(A,B)**, round window membrane model **(C,D)**, 3D model of the inner ear **(E)**, and inner ear model containing round window membrane model (round window membrane model is gray) **(F)**.

**FIGURE 2:**
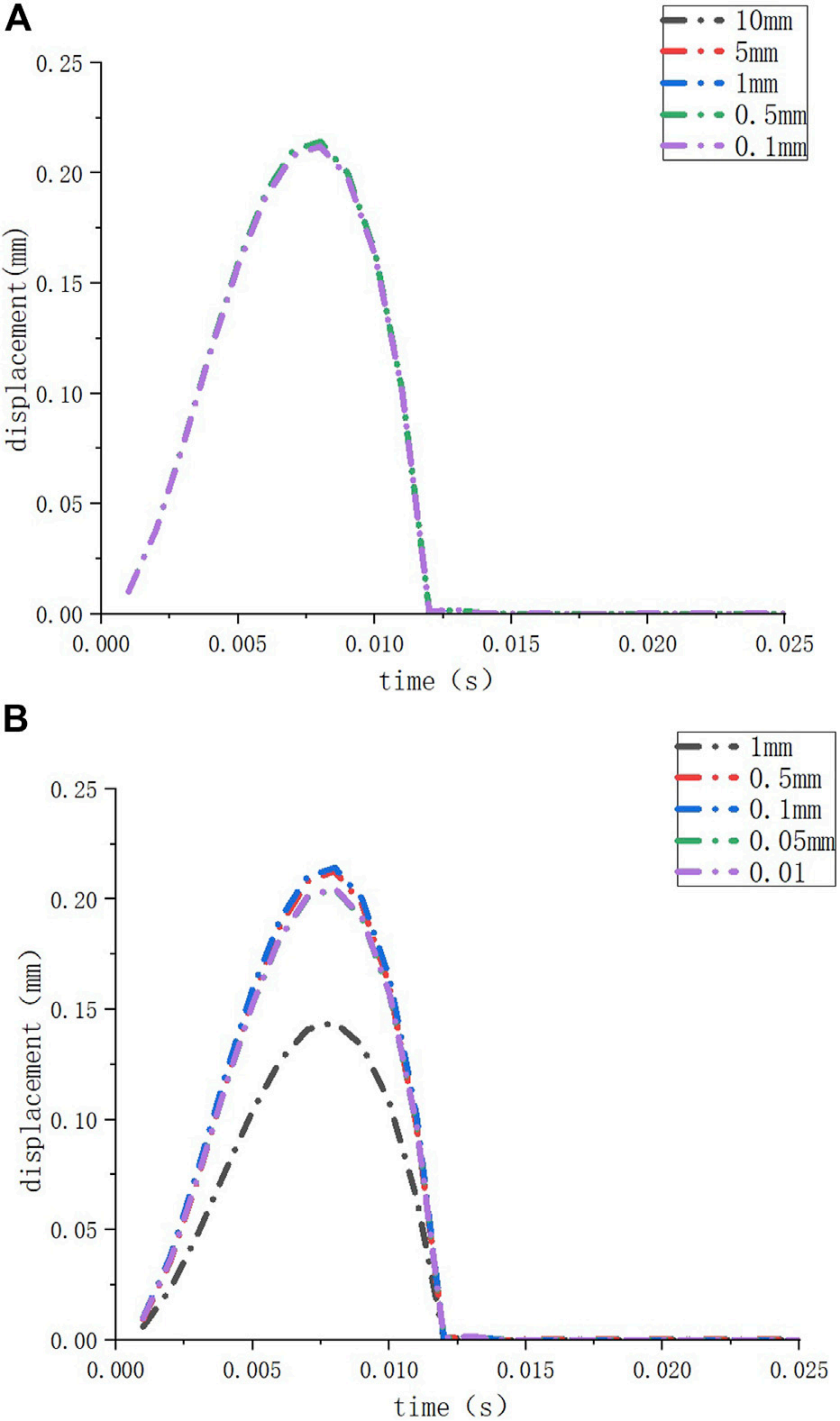
The deformation of the round window membrane when the element sizes of the inner ear model are set to different sizes **(A)**; the deformation of the round window membrane when the element sizes of the round window membrane model are set to different sizes **(B)**.

Inner ear models containing the cochlea, vestibule, semicircular canal, and VA were built using different CT gray values, as shown in Figure 1. The mesh independence was verified for different mesh numbers of the inner ear and round window membrane models, respectively, to exclude the influence of the number of model meshes on the results. The respective results are shown in Figure 2.

We performed mesh independence tests on the inner ear model and round window membrane model to obtain convincing data. We set different element sizes (10 mm, 5 mm, 1 mm, 0.5 mm, and 0.1 mm) for the same inner ear model and set the element size of the round window membrane model at 1 mm. Then, we observed the deformation of the round window membrane under a 10 kPa load for these models (Figure 2A). When the element size of the inner ear model was ≤10 mm (the number of nodes ≥86,313, and the number of elements ≥433,973), the deformations of the round window membranes hardly changed. Similarly, we set different element sizes (1 mm, 0.5 mm, 0.1 mm, 0.05 mm, and 0.01 mm) for the same round window membrane model and set the element size of the inner ear model at 1 mm. Then, we observed the deformation of the round window membrane for these models under a 10 KPa load (Figure 2B). When the element size of the round window membrane model was ≤0.5 mm (the number of nodes >173, and the number of elements >20), the deformations of the round window membranes were close to each other. Considering the calculation time and the accuracy of the results, the element sizes of all inner ear models and round window membrane models were set at 5 mm and 0.1 mm, respectively.

The number of nodes and elements in all models was within the above-specified range. The average round window membrane perimeter was 5.131 mm, and the average round window membrane area was 2.031 mm^2^.

### 3.3 Fluid-solid coupling results

**FIGURE 3:**
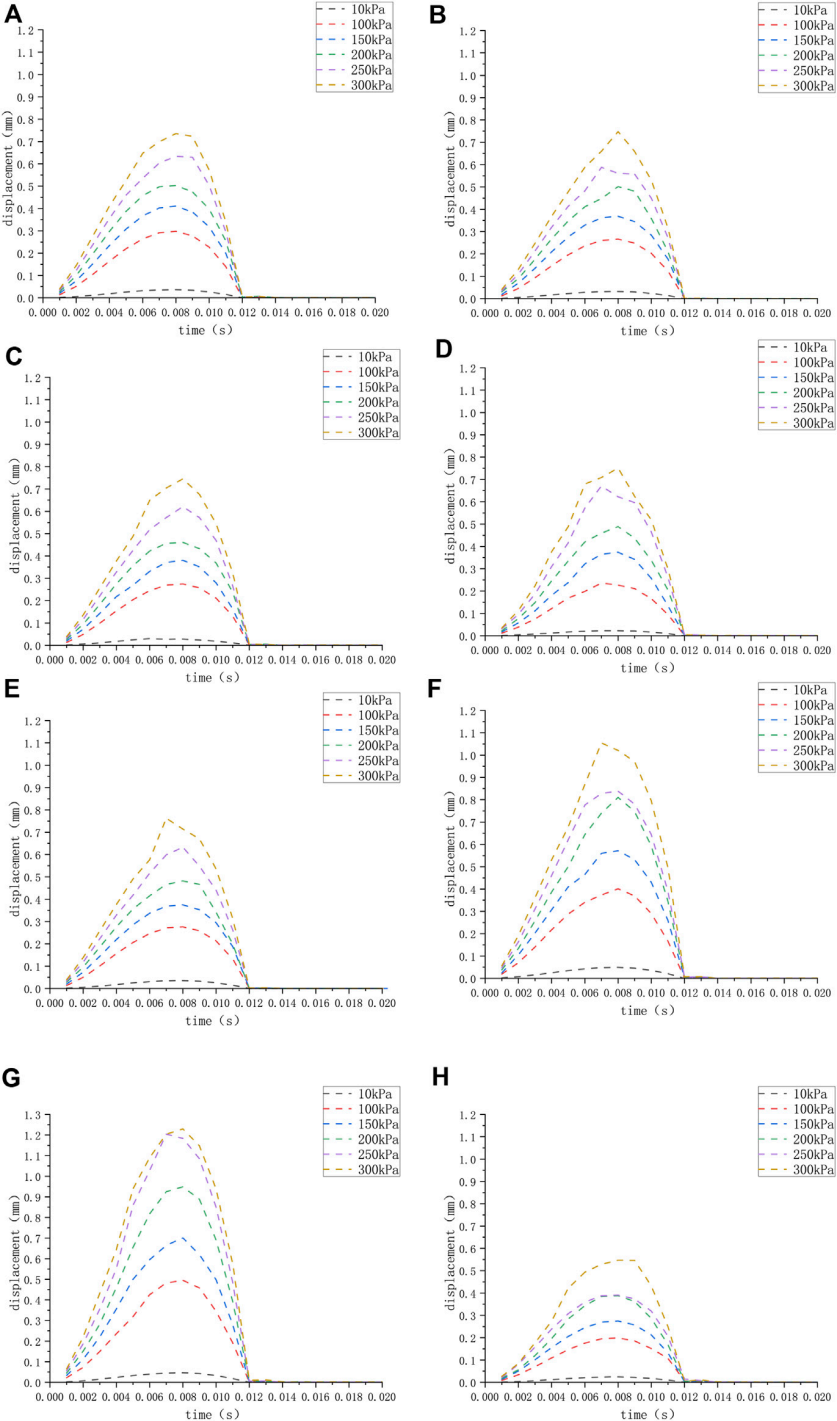
**(H)** The displacement of the round window membrane of the normal inner ear under different pressure loads; **(A–G)** displacement of the round window membrane of the ears **(A–G)** under different pressure loads.

**FIGURE 4:**
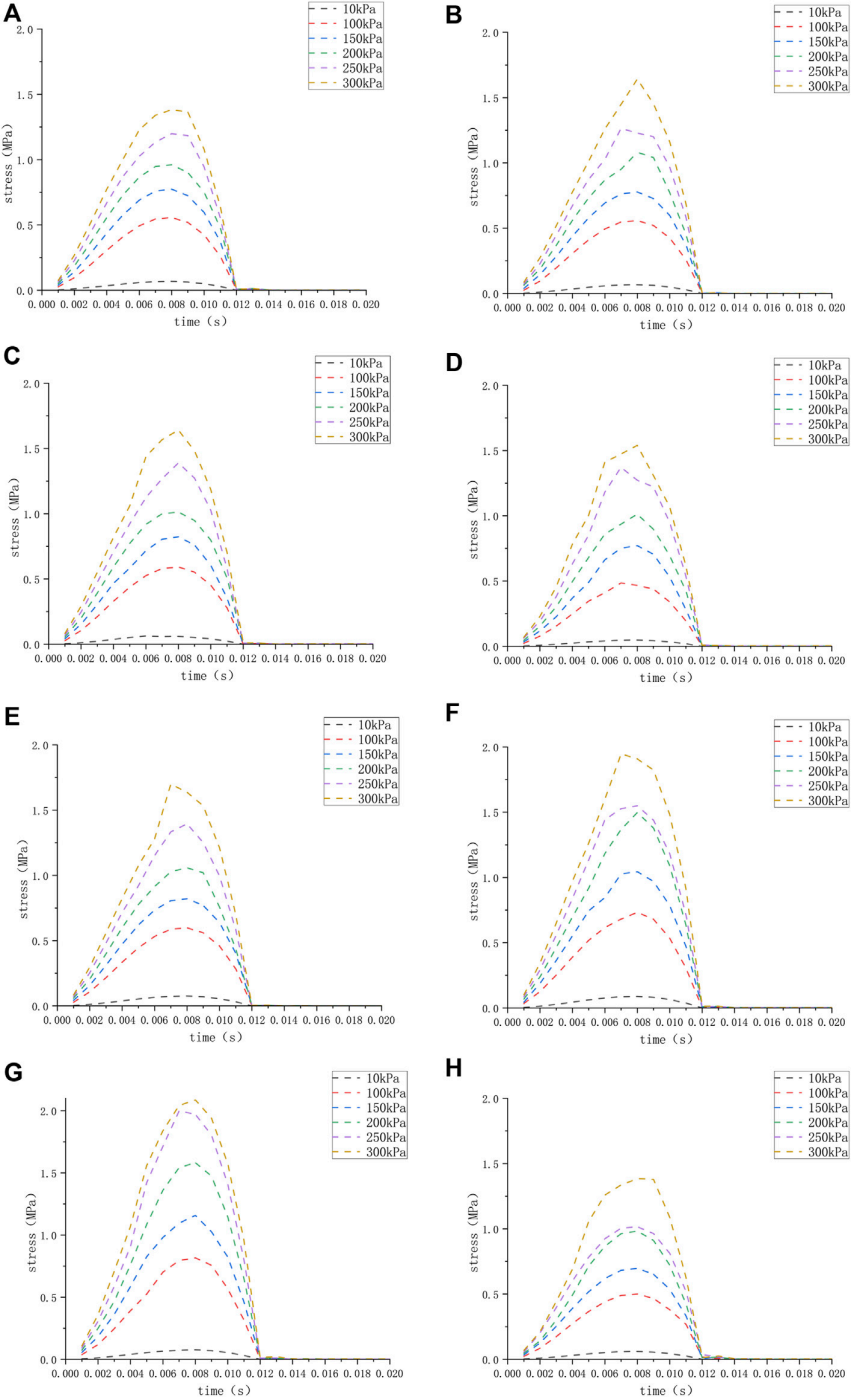
**(H)** The stress of the round window membrane of the normal inner ear under different pressure loads; **(A–G)** the stress of the round window membrane of the ears **(A–G)** under different pressure loads.

We applied a sinusoidal waveform load with a maximum value of 10 kPa, 100 kPa, 150 kPa, 200 kPa, 250 kPa, and 300 kPa to different models. The maximum values of deformation and pressure under different loads for different round window membrane models are shown in Tables 2, 3. The above-stated results were plotted as graphs, as demonstrated in Figures 3, 4. The peak values of maximum deformation and maximum stress for all round window membrane models occurred between 0.007 s and 0.008 s. The deformation and stress increased gently before the peak value and decreased gently after the peak value. Although the load was 0 after 0.012 s, there were still small deformation fluctuations, which could be ignored because of their small order of magnitude.

**TABLE 2:** Maximum values of deformation of different round window membrane models under different pressure load cases.

Inner ear	10 Kpa (mm)	100 KPa (mm)	150 KPa (mm)	200 KPa (mm)	250 KPa (mm)	300 KPa (mm)
Normal	0.024	0.1987	0.2747	0.388	0.3909	0.5468
A	0.0365	0.2977	0.4103	0.5028	0.6338	0.7358
B	0.0322	0.2663	0.3683	0.5015	0.5612	0.7479
C	0.0278	0.275	0.3805	0.4612	0.6208	0.7450
D	0.0227	0.2361	0.3743	0.4889	0.6709	0.7516
E	0.0353	0.2765	0.3750	0.4822	0.6325	0.7628
F	0.0494	0.4011	0.5712	0.8104	0.8383	1.0545
G	0.0461	0.4949	0.7007	0.9487	1.2041	1.2296

**TABLE 3:** Maximum values of stresses on different round window membrane models for different pressure load cases.

Inner ear	10 KPa	100 KPa	150 KPa	200 KPa	250 KPa	300 KPa
	(MPa)	(MPa)	(MPa)	(MPa)	(MPa)	(MPa)
Normal	0.0602	0.5003	0.6962	0.9828	1.0162	1.3851
A	0.0677	0.5568	0.774	0.9605	1.1988	1.3832
B	0.067	0.5588	0.777	1.081	1.2649	1.6427
C	0.06	0.5917	0.8231	1.013	1.3896	1.6398
D	0.047	0.4857	0.7525	1.0126	1.3708	1.5393
E	0.0755	0.599	0.8207	1.0577	1.3949	1.6986
F	0.0884	0.7332	1.0443	1.4973	1.5488	1.9081
G	0.0759	0.8157	1.1581	1.5817	1.9986	2.0865

**FIGURE 5:**
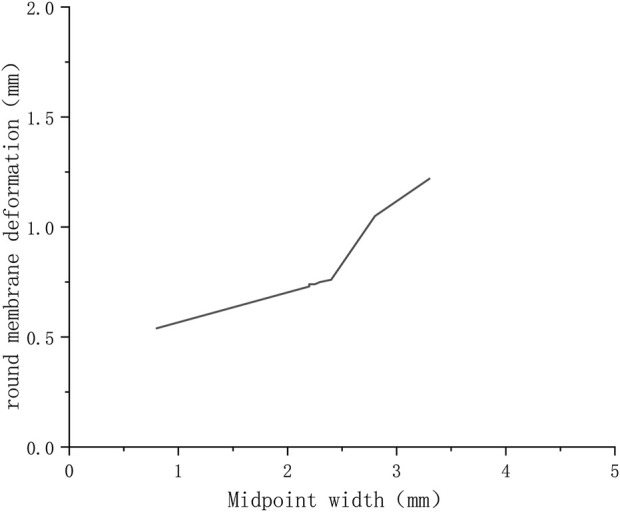
Variation of deformations of round window membranes with widths of VA midpoints (300 KPa).

The maximum deformation for different round window membrane models under 300 kPa is shown in Table 2 and plotted as line graphs in Figure 5. Under the same pressure load, the deformations of the round window membrane gradually increased with the increase in VA midpoint width.

## 4 Discussion

### 4.1 Feasibility of fluid-solid coupling analysis

Fluid-solid coupling, as a cross-discipline between fluid mechanics and solid mechanics, has been variously used in medicine, such as in blood vessels (between the blood and blood vessel walls) and the nasal cavity (between respiratory airflow and the nasal mucosa) (Bie et al., 2020; Athani et al., 2022). In the inner ear, the presence of lymph also makes the flow-solid coupling analysis possible; however, there are fewer studies in this area. In this study, three-dimensional simulation models were created from CT images of LVAS patients, and then fluid-solid coupling numerical simulations were performed to analyze the biomechanical features of the inner ear in LVAS patients and normal people and explain the pathophysiology of LVAS.

### 4.2 The rationality of the model

Because the vestibule window is closed by the stapes footplate, the round window membrane is the most deformable part of the inner ear structure. Changes in lymph can cause deformation of the round window membrane (Shinji Naganawa, Tokiko Koshikawa, Eriko Iwayama, Hiroshi Fukatsu, Tsuneo Ishiguchi, Takeo Ishigaki, Mitsuru Ikeda, Tsutomu Nakashima, and Nobuyasu Ichinose, 2000). Therefore, in this experiment, the round window membranes were used as references to observe the physical quantities, such as deformations and stress caused by the interaction of endolymph during pressure fluctuations, and infer the relevant changes in the inner ear and basilar membrane. Although the round window membrane is not in direct contact with the endolymph, pressure fluctuations of the endolymph can be transmitted to the round window membrane (Takeuchi et al., 1990). Therefore, the models developed in this experiment were reasonable. The pressure lost by the inner ear structures, such as the basilar membrane, during the pressure conduction process was neglected.

In this study, the CT images of the temporal bone could not clearly distinguish the different inner ear fluid spaces, such as endolymph and perilymph. Therefore, this experiment did not distinguish between different inner ear fluid spaces, treating them all as the same. Additionally, previous animal studies have found that the pressure difference between endolymph and perilymph in the inner ear was not significant (Takeuchi et al., 1990). Thus, it is reasonable to set the whole inner ear as having the same fluid.

Recent research has found that LVAS can be better diagnosed by measuring the 3D volume of VA (Boston et al., 2007; Weiss et al., 2022). In this study, LVAS was diagnosed by measuring the width of the VA operculum and midpoint. It was reasonable to use this traditional diagnostic method in our study because there is no unified standard for diagnosing LVAS by VA volume, and we believe that VA width has a greater impact on inner ear pressure than VA volume. However, measuring VA volume is still an interesting proposal. Future studies could explore the influence of VA volume on inner ear pressure and diagnostic criteria of VA volume for LVAS.

### 4.3 The effect of increased cerebrospinal fluid pressure on the inner ear

In this study, all models showed that the larger the pressure load, the larger the deformation and the stress on the round window membrane, and the trend of the deformations and stress on the round window membranes was basically the same as the trend of the load. This indicates that the increase in cerebrospinal fluid pressure will be transmitted to the inner ear through the cerebrospinal fluid-endolymphatic sac-VA-inner ear pathway, and excessive inner ear pressure may cause damage to hair cells and basilar membrane. Okumura et al. have found that excessive pressure fluctuation in the inner ear may even lead to rupture of the round window membrane, causing perilymph fistula (Okumura et al., 1995). Andrews et al. have found that excessive pressure could cause lymph with high protein concentration in the endolymphatic sac to reflux into the inner ear, causing inner ear damage (Andrews, 1997). Rui Li’s study has also found that the larger the load, the greater the deformation of the round window membrane. However, the specific deformation data of the round window membrane were different in this study compared with Rui Li’s one, which might be related to the fact that in the latter study, LVAS inner ear models were artificially created, and the calculation method used was different (Li, 2017).

### 4.4 Effect of VA midpoint width on pressure-limiting effect

On the other hand, by comparing inner ear models with different midpoint widths, we found that the larger the VA midpoint width, the greater the round window membrane deformation, while the stress and deformation on the round window membrane were minimal in the normally developed inner ear model under the same load (Figure 4). This suggested that the smaller the diameter of the VA, the less pressure the inner ear was subjected to. It also demonstrated that VA had a limiting effect on the pressure from the cerebrospinal fluid, and the smaller the VA, the greater its limiting effect. Furthermore, the size of the VA directly affected the stability of the inner ear. Xu Bie et al. have found that VA could buffer the pressure from cerebrospinal fluid and avoid the rapid increase in inner ear pressure, thus, protecting the inner ear tissues through the inner ear model of fluid-solid coupling calculation (Guan et al., 2021). In this regard, we think that the greater the width of the VA midpoint, the more damaged the patient’s inner ear was, which, in turn, led to more severe hearing loss. However, no hearing results were available to support this because the children involved in this study were too young to cooperate in completing the hearing test. A study by Mustafa S. Ascha et al. on 52 patients with LVAS has found that the VA midpoint width correlated with the degree of hearing loss in patients, and for every 1 mm of VA diameter enlargement, the patient’s pure tone hearing threshold increased by 17.5 dB (Ascha et al., 2017). These findings validate the accuracy of our idea.

Because only two patients in this experiment had vertigo symptoms, the relationship between vestibular symptoms and the VA width of LVAS patients could not be verified. The research of Jae-Jin et al. has proven that there were no significant differences between vestibulopathy and non-vestibulopathy groups with regard to the relationship between the development of vestibular symptoms and aqueductal size (Song et al., 2018).

In contrast to LVAS, patients with Meniere’s disease often have stenosis or hypoplasia of the VA and endolymphatic sacs (Wada et al., 2000). A study has found that the angular trajectory of the VA in the axial plane allowed pathological typing of Meniere’s disease into two categories, namely, endolymphatic sac hypoplasia and endolymphatic sac degeneration (Bächinger et al., 2019a; Bächinger et al., 2019b). Our study did not examine inner ear pressure in patients with Meniere’s disease, but we guess that inner ear pressure in patients with Meniere’s disease may not be influenced by cerebrospinal fluid pressure because of stenosis or hypoplasia of the VA and endolymphatic sacs in patients with Meniere’s disease; therefore, inner ear pressure is normal. Sennaroglu et al. have shown that the inner ear pressure in Meniere’s disease patients was not significantly different from the normal population by static acoustic compliance measurements (Sennaroglu et al., 2001). More research is still needed on inner ear pressure in patients with Meniere’s disease.

### 4.5 Effect of inner ear structure on pressure buffering

The maximum load time was 0.006 s, while the maximum deformations of the round window membranes occurred at 0.007–0.08 s, indicating that there was about a 0.001–0.002 s delay in the transmission of pressure from cerebrospinal fluid to the round window membrane. The deformation of the round window membrane cushioned the rapidly increasing pressure in the inner ear and avoided damage to the inner ear basilar membrane and hair cells.

### 4.6 Advantages and limitations

The advantage of this study was that the inner ear models using clinical LVAS temporal bone CT images were reliable, realistic, safe, and inexpensive. Second, using the finite element method, the established models were subjected to fluid-solid coupling analysis, which explained the pathophysiology of LVAS from a new perspective, considering the availability of only a few studies in this area.

This experiment also had some shortcomings. Although the findings of this experiment demonstrated that the enlargement of the VA led to greater pressure on the inner ear, which led to hearing loss, this did not mean that the disease could be cured by narrowing the channel through surgery. The model in this experiment only simulated the pressure transmission of the inner ear, not the physiological function of VA. We guessed that this would be the key to successful treatment, but 3D model simulation could not do this.

LVAS is often accompanied by malformations of the inner ear and the presence of abnormal communication between the perilymphatic space and the subarachnoid space involving the internal auditory canal or the cochlear duct (Sennaroglu and Saatci, 2002; Re et al., 2021). These abnormal communications may have an impact on the pressure of the inner ear, but there is a lack of research in this area. And the effect of these abnormal connections on inner ear pressure was not considered in this study.

In addition, the children involved in this study were too young, and most of them could not cooperate with the hearing test; hence, the relationship between VA width and hearing loss was not studied in this experiment. Future studies may select older children to obtain information on hearing loss.

LVAS is a rare disease. There were few patients in our research institution, and this experiment was a numerical simulation study. Thus, there is no need for too many samples. However, we still look forward to a large sample study in the future.

## 5 Conclusion

CT images of the temporal bone, currently used in clinical practice, could create a complete model of the inner ear containing the VA. By means of fluid-solid coupling analysis, we found that the pressure in the inner ear varied with the cerebrospinal fluid pressure. VA had a limiting effect on the pressure fluctuation from cerebrospinal fluid, and the limiting effect gradually decreased with the expansion of VA. The simulation results of this experiment verified the pathophysiology of LVAS, provided a new idea for the study of LVAS, and laid a foundation for the further study of LVAS through biomechanical methods in the future.

## Data Availability

The original contributions presented in the study are included in the article/supplementary material, further inquiries can be directed to the corresponding author.
